# THE IMPACT OF ADS CLOSURES DURING COVID-19 ON PERSONS WITH DEMENTIA AND THEIR CAREGIVER

**DOI:** 10.1093/geroni/igad104.2659

**Published:** 2023-12-21

**Authors:** Laura Girling, Gretchen Tucker

**Affiliations:** Towson, Towson, Maryland, United States; University of Maryland, Baltimore, Baltimore, Maryland, United States

## Abstract

Due to the COVID-19 pandemic, many Adult Day Services (ADS), community-based programs that provide supervision, social support, and/or assistance with daily activities for clients, either temporarily or permanently terminated in-person services. Such closures left vulnerable older adults with dementia without vital services. To date, there is limited information exploring the nuances and impact of ADS closures on individuals with dementia and their care providers. To address this knowledge gap, data were drawn from an interview-based NIH-funded protocol that evaluated the experiences of informal and formal dementia care providers during the height of the COVID-19 pandemic. Data were filtered by the subsample (n=25) who provided care to an individual with dementia who participated in an ADS program. Analysis focused on all data coded “ADS,” which encompassed discussion pertaining to adult day programming. Content analyses indicated a range of themes including 1.accelerated cognitive and psychological decline, 2.engaging is precarious conduct, 3.increased caregiver burden (financial, physical, emotional), and 4.premature transitions to institutional care settings. Despite well intentioned efforts to shield the older adult population from harmful exposures during the height of the pandemic, ADS closures (both permanent and temporary) negatively impacted the health and welfare of an already vulnerable population and their caregivers. Findings add to the ongoing literature suggesting ADS and analogous supports should be classified as essential services and not face closure mandates during similar public health emergencies.

